# Supplementary material for: Light sheet fluorescence microscopy of cleared human eyes
Source: Commun Biol. 2023 Oct 10;6:1025. doi: 10.1038/s42003-023-05401-0 (PMC10564773; doi:10.1038/s42003-023-05401-0)
Supplement: Supplementary file 2 — Supplementary Information [file 42003_2023_5401_MOESM2_ESM.pdf]

Supplementary data

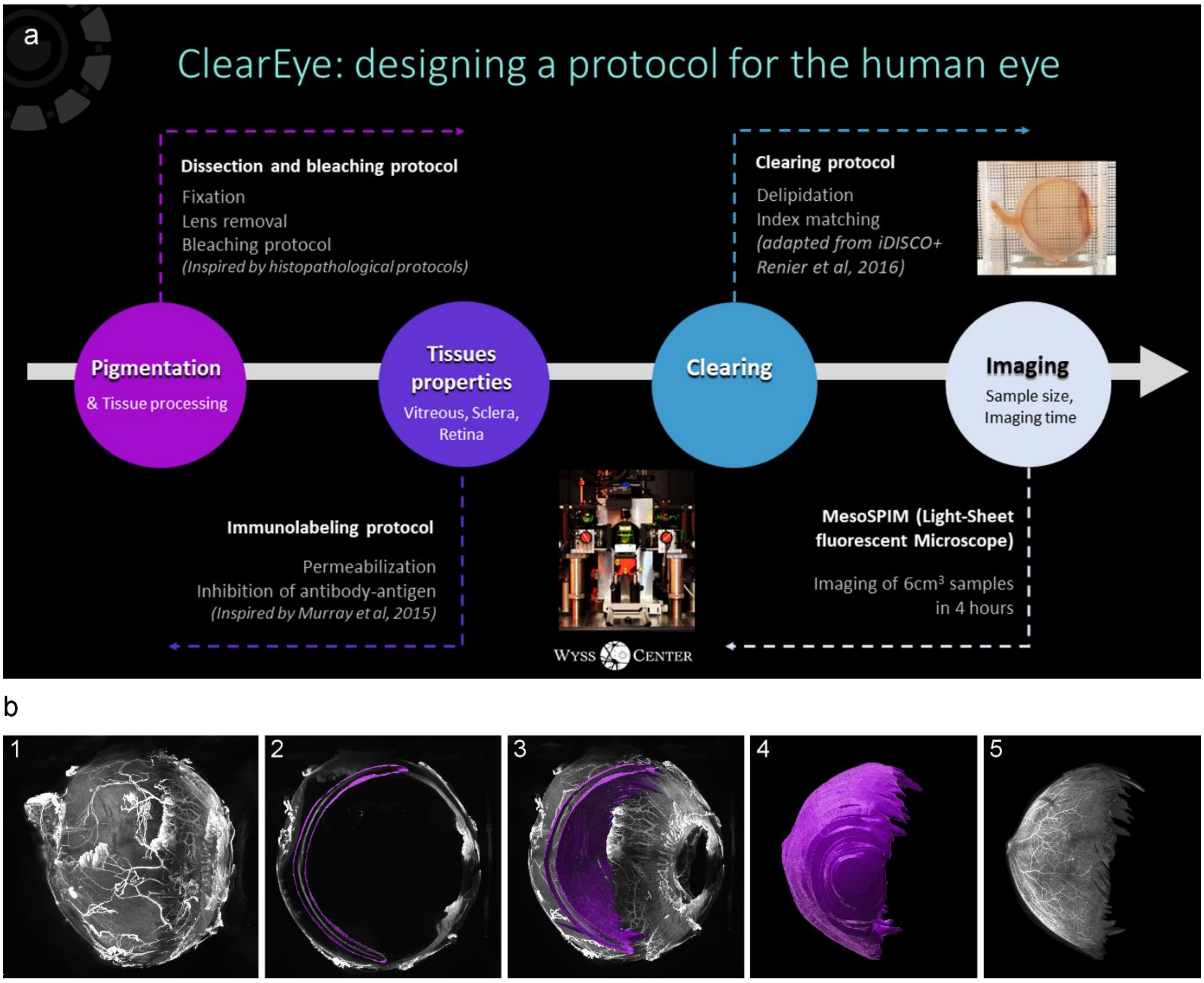

Supplementary Figure 1: a: Schematic of key steps developed for the ClearEye protocol to address technical challenges in imaging an entire labeled human eye. b: An example of manual segmentation for 3D rendering of selected target using Imaris software (Bitplane). Here retina isolation is shown. b, 1: Whole unsegmented 3D sample. b, 2: Plane by plane manual isolation of the retina using tracing a mask (in purple) around the region of interest. b, 3: 3D rendering of the mask traced on the entire sample after automatic 3D reconstruction by the software. b, 4: 3D aspect of the mask once finished and isolated from the sample. b, 5: Isolation of the object inside the mask, here the retina.

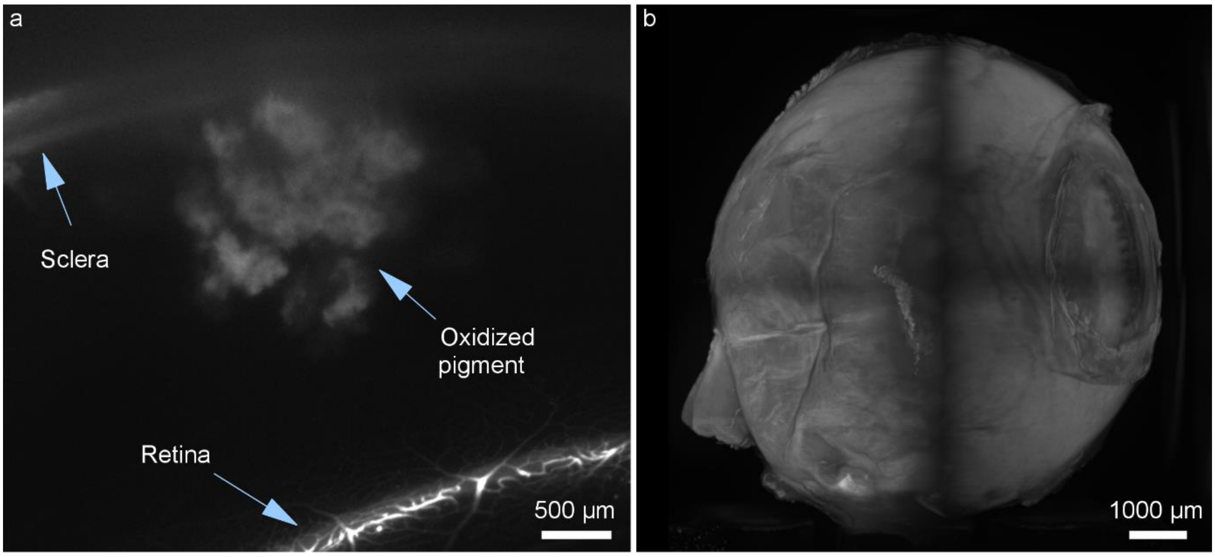

Supplementary figure 2: Artifacts encountered. a: Oxidized pigment located in subretinal space between sclera and retina (ColIV labeling, excitation wavelength: 647 nm). b: Background autofluorescence emitted with 488 nm excitation.

Panel	Donor's age	Labeling	Primary Antibody reference	Secondary antibody reference	Wavelenght	Zoom	Pixel size	Step size	Manual segmentation (Imaris software)
**1D**	84 yo	Collagen IV	134001, Biorad	A-21447, Thermofisher	647	0.8X	8.235µm	5µm	None
**1E**	84 yo	Collagen IV	134001, Biorad	A-21447, Thermofisher	647	0.8X	8.235µm	5µm	None (section with crop tool)
**1F**	84 yo	Collagen IV	134001, Biorad	A-21447, Thermofisher	647	0.8X	8.235µm	5µm	Isolated retina
**1G**	84 yo	Alpha-SMA	A2547, Merck	A-21203, Thermofisher	561	0.8X	8.235µm	5µm	Isolated choroid
**1H**	84 yo	Collagen IV	134001, Biorad	A-21447, Thermofisher	647	0.8X	8.235µm	5µm	Isolated retina and crops
**1I**	84 yo	Collagen IV	134001, Biorad	A-21447, Thermofisher	647	1.25X	5.26 µm	5µm	Isolated retina
**1J**	84 yo	Collagen IV	134001, Biorad	A-21447, Thermofisher	647	1.25X	5.26 µm	5µm	Orthoslicer
**1K**	84 yo	Collagen IV	134001, Biorad	A-21447, Thermofisher	647	1.25X	5.26 µm	5µm	Orthoslicer
**1K**	84 yo	Alpha-SMA	A2547, Merck	A-21203, Thermofisher	561	1.25X	5.26 µm	5µm	Orthoslicer
**2A**	89 yo	Tubuline III	T2200-200uL, Merck	A-31573 Thermofisher	647	1X	6.55µm.	5µm	None (section with crop tool)
**2B**	16 yo	Tubuline III	T2200-200uL, Merck	A-31573 Thermofisher	647	0.8X	8.23µm	5µm	None (section with crop tool)
**2C**	89 yo	Tubuline III	T2200-200uL, Merck	A-31573 Thermofisher	647	0.8X	8.23µm	5µm	None (section with crop tool)
**2D**	47 yo	Tubuline III	T2200-200uL, Merck	A-31573 Thermofisher	647	0.63X	10.52 µm	5µm	None (section with crop tool)
**2E**	47 yo	Collagen IV	134001, Biorad	A-21447, Thermofisher	647	0.63X	10.52 µm	5µm	Orthoslicer
**2F**	89 yo	Tubuline III	T2200-200uL, Merck	A-31573 Thermofisher	647	3.2X	2.03µm	3µm	Orthoslicer
**2G**	89 yo	Tubuline III	T2200-200uL, Merck	A-31573 Thermofisher	647	3,2X	2.03µm	3µm	3D rendering of Schlemm's canal
**2H**	47 yo	Collagen IV	134001, Biorad	A-21447, Thermofisher	647	0.63X	10.52 µm	5µm	None
**2H**	47 yo	Tubuline III	T2200-200uL, Merck	A-31573 Thermofisher	647	0.63X	10.52 µm	5µm	None
**2I**	47 yo	Tubuline III	T2200-200uL, Merck	A-31573 Thermofisher	647	0.63X	10.52 µm	5µm	None
**2J**	84 yo	Collagen IV	134001, Biorad	A-21447, Thermofisher	647	2X	3.30µm	3µm	None
**2J**	84 yo	Alpha-SMA	A2547, Merck	A-21203, Thermofisher	561	2X	3.30µm	3µm	None
**2K**	40 yo	Collagen IV	134001, Biorad	A-21447, Thermofisher	647	0.63X	10.52 µm	5µm	3D rendering of Ciliary artery

Supplementary table

Patient’s age, antibodies used, technical imaging characteristics and post-acquisition manipulation for each panel of the main figures.

Supplementary video

3D rendering of the samples described in the main figures.

Uploaded on Communications Biology website.
