# Supplementary material for: Light sheet fluorescence microscopy of cleared human eyes
Source: Commun Biol. 2023 Oct 10;6:1025. doi: 10.1038/s42003-023-05401-0 (PMC10564773; doi:10.1038/s42003-023-05401-0)
Supplement: Supplementary file 3 — Description of Additional Supplementary Files [file 42003_2023_5401_MOESM3_ESM.pdf]

Description of Additional Supplementary Files

File name: Supplementary Video

Description: 3D rendering of the samples described in the main figures.
